# Low rate of detection of SARS-CoV-2 RNA in deceased tissue donors

**DOI:** 10.1007/s10561-022-10054-0

**Published:** 2022-12-09

**Authors:** Melissa A. Greenwald, Eduard Grebe, Valerie Green, Alyce Linthurst Jones, Jeffrey M. Linnen, Phillip Williamson, Michael P. Busch, Matthew J. Kuehnert

**Affiliations:** 1 Donor Alliance, Denver, CO USA; 2 Uniformed Services University of the Health Sciences, Bethesda, MD USA; 3 MA Greenwald Consulting, Chicago, IL USA; 4 Vitalant Research Institute, San Francisco, CA USA; 5 University of California San Francisco, San Francisco, CA USA; 6 DSI-NRF Centre of Excellence in Epidemiological Modelling and Analysis, Stellenbosch University, Stellenbosch, South Africa; 7 Creative Testing Solutions, Tempe, AZ USA; 8 LifeNet Health, Virginia Beach, VA USA; 9 Grifols Diagnostic Solutions, San Diego, CA USA; 10 Musculoskeletal Transplant Foundation, Edison, NJ USA; 11 Hackensack Meridian School of Medicine, Hackensack, NJ USA

**Keywords:** Tissue donor, Organ donor, Infection, Viremia, RNAemia, SARS-CoV-2

## Abstract

Given the possibility for disease transmission, this study was performed to determine whether there is detectable SARS-CoV-2 viral RNA in the blood of deceased tissue donors. A retrospective analysis of blood samples from eligible deceased tissue donors from Oct 2019 through June 2020 was performed. Plasma aliquots were initially tested with a SARS-CoV-2 NAT Assay; positive samples were further tested using an alternate NAT and an antibody assay. The proportion of donors with confirmed RNAemia and 95% confidence intervals were computed. Of donor samples collected in 2019, 894 yielded valid results, with 6 initially positive, none of which confirmed positive by alternate NAT. Of donor samples collected in 2020, 2562 yielded valid initial NAT results, with 21 (0.8%) initially positive. Among those, 3 were confirmed by alternate NAT, 17 were not confirmed, and 1 had an invalid alternate NAT result. The rate of SARS-CoV-2 RNAemia in deceased tissue donors is approximately 1 per 1000, and it is unknown whether this RNAemia reflects the presence of infectious virus. Given these results, the risk of transmission through tissue is thought likely to be low.

## Introduction

Since the COVID-19 pandemic was declared in March 2020, much has been learned, and much remains to discover about COVID-19 and its cause, SARS-CoV-2. As of July 3, 2022, more than 549 million people worldwide are confirmed to have been infected, and more than 6 million have perished (Johns Hopkins University Medicine 2022). In the United States, the death toll from COVID-19 has surpassed that of the 1918 Flu Pandemic, killing over 1 million Americans, with numbers increasing daily. Two years after SARS-CoV-2 was first identified, transmission has not subsided, variants with increased transmissibility have emerged, and the virus appears to be detectable beyond the respiratory tract. Further, damage from COVID-19 has been observed in a multitude of tissues and organs such as the brain, olfactory nerves, myocardium, kidneys, arteries, and intestines, although live viral effect has not been proven (Xu et al. 2021).

It is imperative to understand the possible risks of transmission via tissue and organ transplantation. After reports indicated that SARS-CoV-2 was detectable in blood by nucleic acid amplification testing (NAT) in mildly ill or asymptomatic individuals, including in asymptomatic blood donors (Huang et al. 2020; Bakkour et al. 2021), there was concern that viremia (if present) could result in transmission through transfusion and transplantation, because many transfusion-transmitted infections (TTIs) are also transmitted via transplantation. While in vitro infectivity studies have not yet been performed using NAT positive blood samples, the presence of RNA in the blood (i.e., RNAemia) raises concern of potential transmission through tissue and organ transplantation. Since that time, autopsy and other studies have detected viral RNA and visualized intact viral particles in multiple organs, including kidneys, the gastrointestinal tract, male genitourinary tract, and amniotic membrane (Bradley et al. 2020; Best et al. 2021; Gaussen et al. 2021; Penfield et al. 2020; Trypsteen et al. 2020).

Tissue donors are evaluated for communicable disease risks to minimize potential transmission associated with tissue transplantation. It is not known at this time how viable SARS-CoV-2 is when located outside the respiratory tract, the frequency or length of viremia, and how frequently the virus is found in, or whether transmissible by, tissues outside the respiratory tract. Given the challenges with identifying viral infection based solely on symptoms and the transmission of this virus from asymptomatic individuals, we conducted this study to determine whether there is detectable viral RNA in the blood of deceased tissue donors. We sought to gauge the risk of SARS-CoV-2 transmission through tissue by detecting blood RNAemia of eligible deceased tissue donors. American Association of Tissue Banks’ (AATB) recommendations in effect during most of the study period indicated that in order to donate, donors should be free of symptoms of SARS-CoV-2 infection and not have had close contact with a person who had confirmed infection, in addition to the traditional screening for health and social behaviors that are exclusionary (American Association of Tissue Banks 2020). This study evaluating the presence of RNA-emia in deceased tissue donors (“donor/s”) is a first step in developing data regarding the risk of SARS-CoV-2 viremia in accepted tissue donors, needed to inform prudent policy on donation.

## Materials and methods

**Fig. 1:**
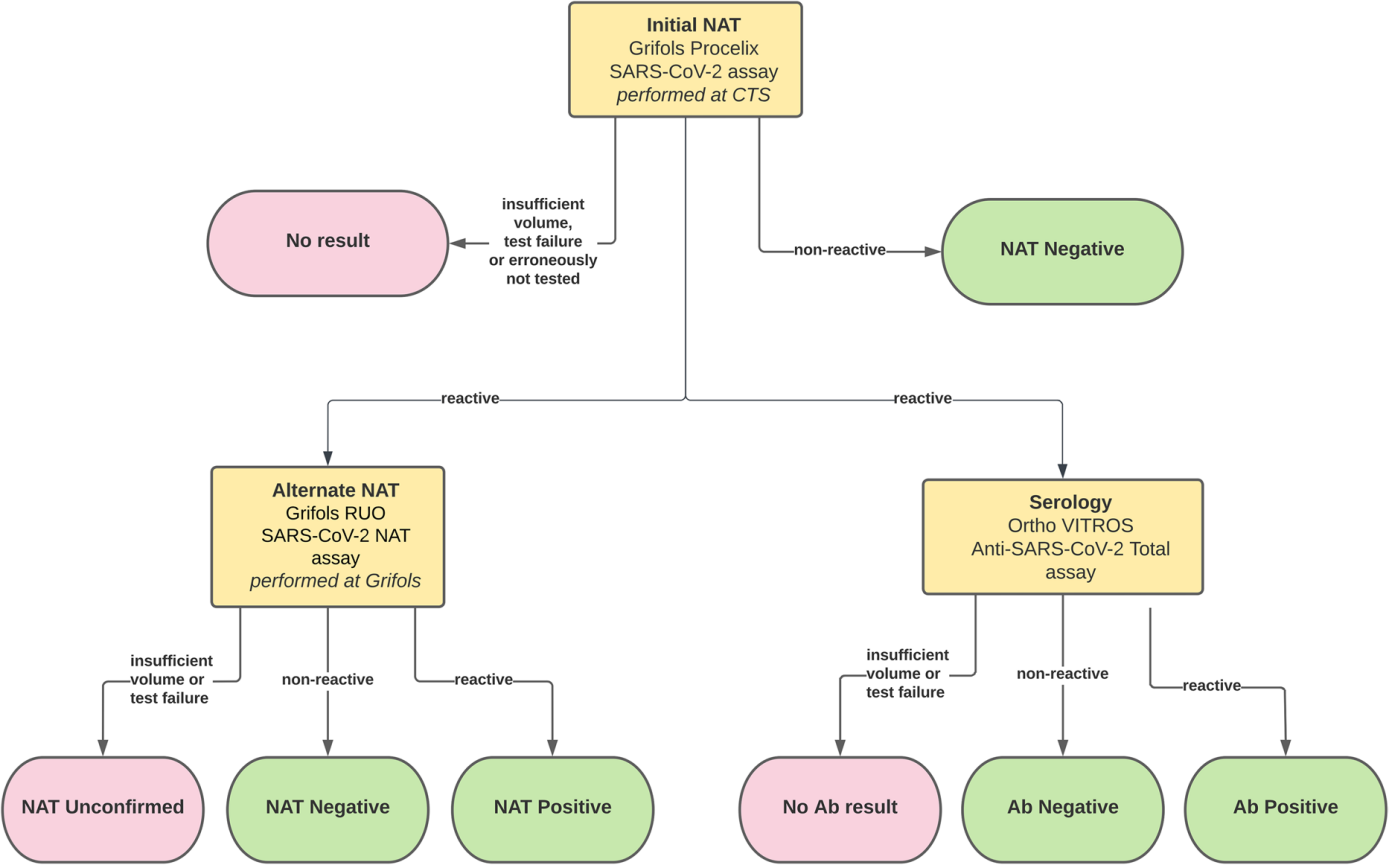
Testing Algorithm Overview. Initial NAT was performed first. If initial NAT yielded no test results, the specimen result is reported as “No result” and no further testing was performed. If the initial NAT test was nonreactive, the specimen result is reported as “NAT Negative” and no further testing was performed. Initial NAT-reactive specimens were further tested with an alternate NAT to determine the final NAT disposition. NAT results are reported as “NAT Unconfirmed” if there was not an alternate NAT result, “NAT Negative” if the alternate NAT was non-reactive,” and “NAT Positive” if the alternate NAT result was reactive. Initial NAT-reactive specimens were also tested for antibodies to SARS-CoV-2. Antibody results were reported as “No Ab result” if there was insufficient volume to perform testing or a test failure, “Ab positive” if the assay was reactive, and “Ab negative” if the assay was nonreactive. Initial NAT and antibody testing performed at Creative Testing Solutions (CTS), and alternate NAT performed at Grifols

Participating tissue banks with donors from multiple areas of the US and Canada provided a de-identified list of all available deceased donor specimens collected and retained frozen during the study period (October 1, 2019, to June 30, 2020). Because specimens were de-identified, it is not possible to know how many donors were both organ and tissue donors, whether specimens were collected before or after death (the study assumes worst-case-scenario that specimens were collected after death), and no results were provided to participating tissue banks. Specimens were considered for inclusion according to study criteria (Table 1), and then selected for testing as follows. For specimens collected during 2019 (control arm, tested to estimate the baseline assay specificity during a time when the pre-test probability of SARS-CoV-2 was anticipated to be zero), all specimens were included from four tissue banks that had fewer than 100 specimens available, while a random selection was made from available specimens of the remaining tissue banks to target a total sample size of 1000 for 2019. The testing algorithm (Fig. 1) in this study relied on a screening NAT assay, confirmation of initial positive samples by an alternate NAT assay (performed by Grifols, San Diego, CA) to determine final NAT disposition, and when adequate sample was available, also testing initial positives for anti-SARS-CoV-2 antibodies. Initial NAT and antibody testing were performed by Creative Testing Solutions (CTS) (Tempe, AZ).

**Table 1:** Study criteria

Inclusion criteria	Exclusion criteria
Donations collected within study timeframe	Donation occurred outside the study timeframe
a. October 1–December 31, 2019 (control group)	
b. January 1–June 30, 2020 (study group)	
Donor determined eligible for donation according to FDA regulatory requirements^1^	Donor determined ineligible according to FDA regulatory requirements, or donor known or suspected to have SARS-CoV-2 (including positive nasopharyngeal swab specimens)
Donor authorized for research	Research authorization not obtained
Archive specimens available with sufficient specimen and volume type: minimum of 1.75 mL total sample, of which > 1 mL is plasma collected in EDTA tubes from deceased donors—the remainder of the specimen volume may be provided using serum specimens	Insufficient sample volume
Donations collected within the United States or Canada^2^	Donor demographic data not provided
*Unknown (deidentified donors)*	
Whether the donor also was an organ donor	
Exact timing of specimen collection—specimens must be collected within 7 days of tissue collection and meet IFU specimen requirements for donor screening assays; the vast majority of non-organ donor specimens would have been collected within hours after cessation of the heartbeat, and specimens from brain-dead organ donors are typically collected within 2–3 days prior to donation (i.e., prior to cessation of heartbeat)	

^1^While it is not possible to know whether donors in 2020 had nasopharyngeal swab samples tested for SARS-CoV-2 RNA, any donors who were tested had negative results and no symptoms of COVID because tissue establishments voluntarily excluded known or suspected SARS-CoV-2 (+) donors

^2^Convenience sample based on willingness of tissue establishment to participate in the study

**Fig. 2:**
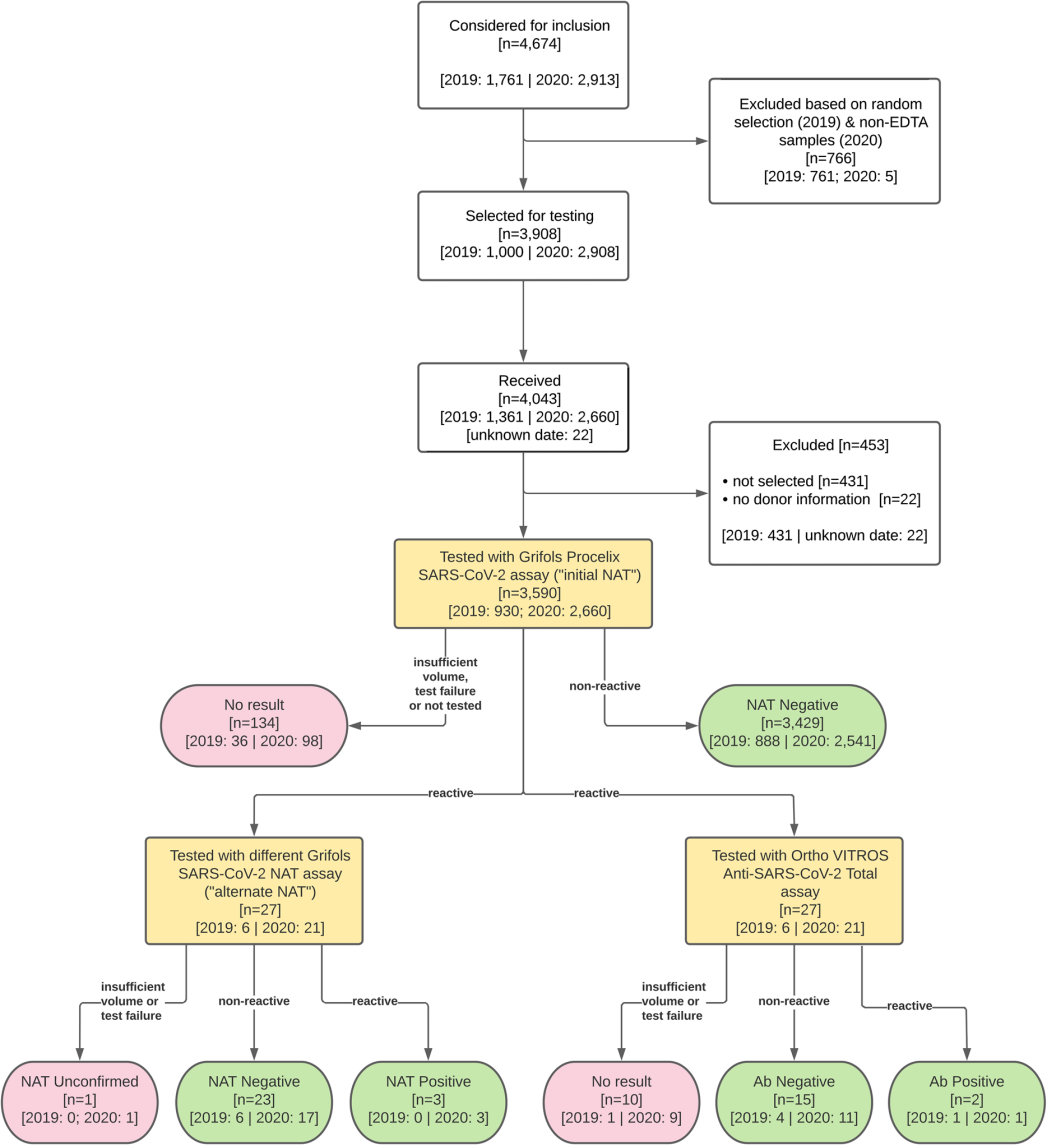
Study algorithm. Summarizes all specimens considered, disposition, and results

After excluding ineligible samples sent to the testing laboratory in error, 930 2019 samples were tested, and 894 had valid test results and were included in the analysis. All specimens collected in 2020 meeting eligibility criteria were tested (2660), and all 2561 specimens that yielded valid test results were included in the analysis. Samples without test results either had insufficient volume, a test failure occurred, or was erroneously not tested (Fig. 2).

Archive donor specimens in aliquot tubes accessed for this study had been stored at − 30 to − 35 °C. Donor specimens were shipped to CTS on dry ice, and remained frozen at ≤ 20 °C until it was time for specimens to be aliquoted for testing. Specimens were prepared for testing by thawing them, aliquoting them into appropriate low volume testing tubes labeled with unlinked sample ID, and refrozen at ≤ 20 °C until being shipped on dry ice to another CTS site that performed testing. Samples were then thawed (second freeze/thaw cycle), centrifuged, and then routed for testing.

Aliquots labeled as plasma (originally collected in EDTA) were tested with the Grifols Research Use Only (RUO) Procleix SARS-CoV-2 Assay on the Procleix Panther System (“screening NAT”) (Bakkour et al. 2021; Sauleda et al. 2022; Grifols 2022). The Procleix SARS-CoV-2 Assay is a qualitative in vitro nucleic acid test that uses transcription mediated amplification (TMA) for the detection of SARS-CoV-2 nucleic acid. The assay is a highly sensitive and specific test for the detection of SARS-CoV-2 RNA on a high-throughput, automated platform. An evaluation of the performance characteristics can be found in Sauleda et al. (2022). Results obtained from negative and positive calibrators are used to determine the validity of the run and to establish the assay cutoff values for the internal control signal and the analyte signal. Reactive or nonreactive results for each specimen tested are provided by the instrument based on predetermined calculations of the cutoff values that are coded within the assay software. A single test was performed on each sample and results were evaluated for reactivity. Aliquots from donors with reactive initial NAT results were sent to Grifols for “alternate NAT” testing using Grifols Research Use Only (RUO) SARS-CoV-2 NAT, a lab-developed supplemental TMA assay that targets a different region of the SARS-CoV-2 genome, to verify reactivity (Sauleda et al. 2022). The “alternate NAT” assay is a qualitative in vitro nucleic acid test with demonstrated comparable sensitivity to the “screening NAT” (Sauleda et al. 2022). For some initial reactive donations, serum aliquots were used for the alternate assay due to insufficient residual plasma volume. A single sample was not routed for alternate NAT testing due to insufficient volume. Reactive samples (plasma or serum aliquots), if volume available, were also routed for antibody testing using the Ortho Clinical Diagnostics EUA VITROS Immunodiagnostic Products Anti-SARS-CoV-2 Total test. The Ortho Clinical Diagnostics VITROS^®^ platform uses immunometric technology for qualitative measurement of total antibody (IgG and IgM) to SARS-CoV-2. All results were submitted to Vitalant Research Institute (VRI) for further evaluation.

Specimen collection date, the donor’s age in years at death, sex, race/ethnicity, zip code of residence, and state/province were collected in deidentified form, i.e., linked only to the tested specimens. The proportion of donors with confirmed blood RNAemia was computed and Wilson score 95% confidence intervals are reported.

## Results

The study population is not representative of the demographics of the general population of the United States or Canada, with the study population heavily skewed towards male (68.2%) and white (69.6%) tissue donors. Hispanic ethnicity could be ascertained for a small minority of donors in the study. Although all Census regions of the United States and the Canadian province of Nova Scotia are represented, a large majority of donors resided in the South (64.8%) (Table 2).

**Table 2:** Demographic breakdown of deceased tissue donors included in the study

	Group	*n* (%)
Sex	Male	2356 (68.2)
	Female	1100 (31.8)
	Total	3456 (100.0)
Race	American Indian and Alaska Native	14 (0.4)
	Asian	41 (1.2)
	Black or African American	351 (10.2)
	Native Hawaiian and Other Pacific Islander	2 (0.1)
	White	2404 (69.6%)
	Other/Unknown	644 (18.6)
	Total	3.456 (100.0)
Ethnicity*	Hispanic	193 (5.6)
	Non-hispanic	530 (15.3)
	Unknown	2733 (79.1)
	Total	3.456 (100.0)
Geographic Region	Northeast (US)	256 (7.4)
	Midwest (US)	451 (13.0)
	South (US)	2240 (64.8)
	West (US)	292 (8.5)
	Novia Scotia (CA)	54 (1.6)
	Unknown	163 (4.7)
	Total	3.456 (100.0)

All samples with valid initial NAT results are included in Table 2, including the single sample that failed to yield a valid alternate NAT result. While donors having no demographic data available were excluded from the study, some donors included in the study had some missing demographic data

*The degree of missing data on Hispanic ethnicity is so great that no inference about the representation of Hispanic donors in the study should be made

As represented in Fig. 2, a total of 3455 unique donor samples provided evaluable, valid data, i.e., a non-reactive initial NAT result or a reactive initial NAT result *and* a valid alternate NAT result; 134 additional samples did not yield valid screening NAT results (Table 3), and one was initially reactive but unconfirmed because alternate NAT did not yield a valid result. This sample was kept in the descriptions of the study population, but was not in the computation of proportions reactive. Of the 3455 donor samples with valid initial and alternate NAT results, 894 were collected in the last quarter of 2019 and 2561 during the first half of 2020.

**Table 3:** Potential reasons for invalid NAT assay results

Error type	Example
Sample inhibitory substances	Cadaveric blood specimens, when tested neat, may be invalid due to inhibitory substances within the specimen
Chemistry error	Internal Control Signal lower than the Internal Control cutoff
Assay processing error	Clot detected in sample
	Reagent dispensing error
Results processing error	RLU value outside of software range
	Invalid calibrator

Twenty-seven samples that were reactive on the initial NAT were routed to Grifols for alternate NAT testing. For 7 of these, serum aliquots were used due to insufficient residual plasma volume for the alternate assay, and one sample had insufficient volume for alternate NAT testing.

Of the 894 unique donor blood samples collected in 2019 yielding valid initial NAT results, 6 were initially reactive, and 888 non-reactive. None or 0.00% were confirmed reactive for SARS-CoV-2 RNA (95% confidence interval [CI] 0.00–0.43%); one sample did not confirm reactive but did have a positive Ab result. Of the 2562 unique donor samples collected in 2020 yielding valid initial NAT results, 21 were initially reactive, and 2541 non-reactive. In alternate NAT testing, three or 0.12% (95% CI: 0.04–0.34%) were confirmed RNAemic, 1 was yielded an invalid alternate NAT result (therefore disposition of “NAT unconfirmed”), and the remainder were nonreactive.

Among all donor samples tested by initial NAT (including both 2019 and 2020), 125 samples eligible for inclusion had insufficient volume for initial NAT testing, 5 yielded an invalid initial NAT test result (test failure), and four samples were erroneously not tested using initial NAT. A further sample from 2020 tested initially reactive, but alternate NAT testing yielded an invalid result. Because of small sample size, donor demographics for positive samples are not being reported.

Of all 27 initially NAT reactive samples, 17 had sufficient volume available for antibody testing using the Ortho assay. One confirmed NAT positive sample (2020), and one initially reactive sample that was not confirmed positive by alternate NAT (2019), were positive for anti-SARS-CoV-2 Spike antibodies.

**Fig. 3:**
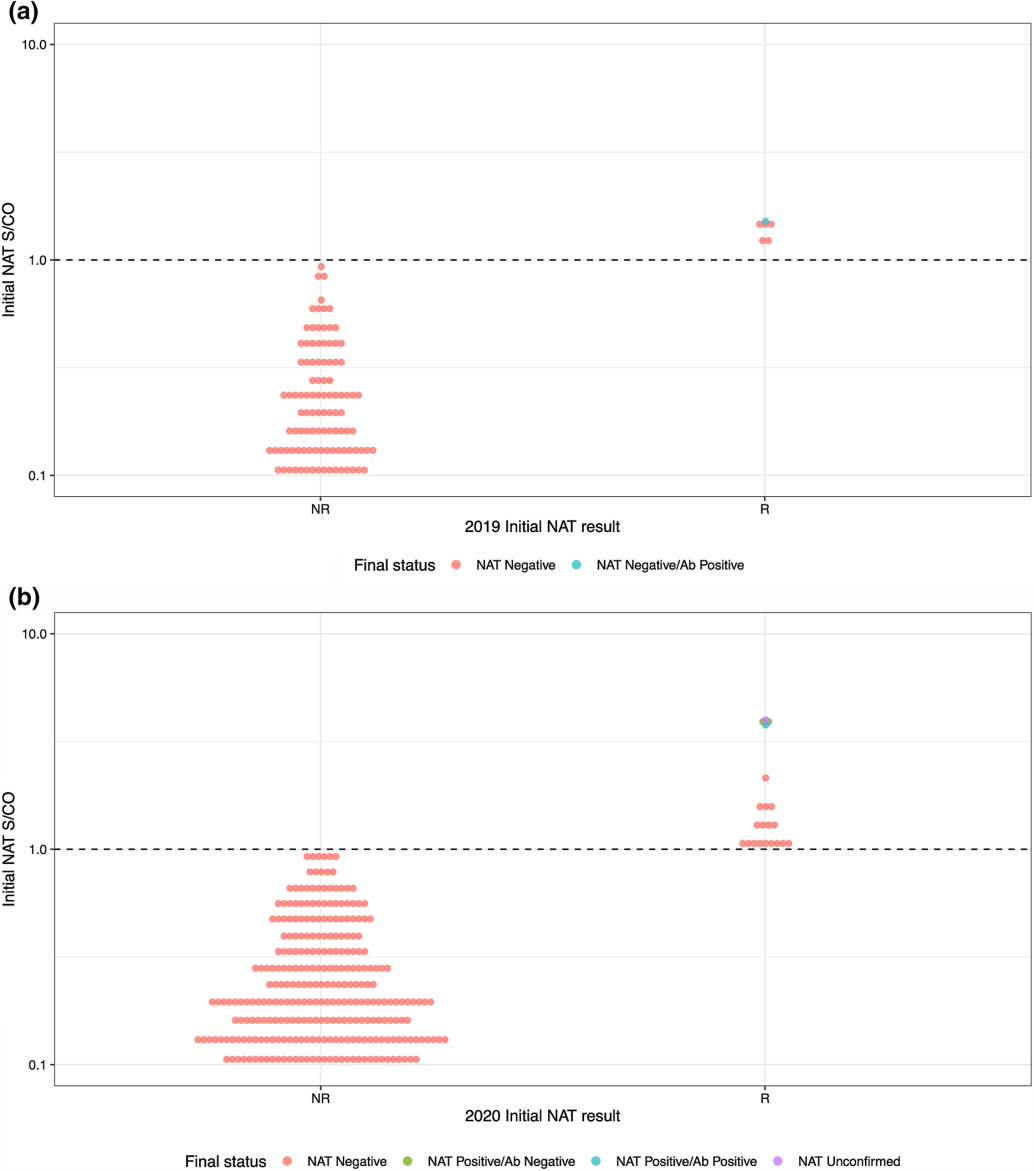
S/CO values for all Initial NAT results compared to final test results. **A** represents 2019 specimen results, while **B** represents 2020 specimen results. The *y* axis represents the Signal/Cut Off (S/CO) ratio values obtained on the initial NAT assay. The x axis organizes S/CO values among initial NAT reactive (R) vs initial NAT non-reactive (NR), while also indicating (by color, as labeled) both the final NAT disposition and the Antibody results. See Fig. 1 for results description. The S/CO cutoff between positive and negative results is 1.0. Positive results close to the assay cutoff generally have a higher likelihood of being false-positive than those with higher S/CO values

Figure 3 shows the distribution of signal-to-cutoff ratios (S/COs) obtained using the screening NAT assay, and final status based on alternate NAT and serology. The confirmed NAT positive samples all had high S/COs in initial NAT testing relative to the S/COs of initially reactive samples that were not confirmed. The figure includes the single sample that did not have a valid alternate NAT result.

## Discussion

This is the first known study publishing data regarding the presence of SARS CoV-2 RNA in the blood of deceased tissue donors. Between January 1 and June 30, 2020, among the included tissue donors (who were not suspected at the time of having COVID-19 and no vaccine was available), there were 3 confirmed positive donor samples by alternate NAT testing among 26 initial positives that had a confirmatory test result, about 0.12% of donors tested. Given the relatively high S/CO values of the initial positive results, that then also confirmed positive on the alternate NAT, and high specificity of the confirmed assays, our data support the strong likelihood that the 3 positive confirmed samples represent RNAemia. However, this is a low confirmation rate and possible causes for lack of confirmation include interfering substances in deceased donor plasma, issues related to sample quality after storage, differences in assay sensitivity, or other/unresolved assay issues. It should be noted that we observed a higher initial NAT reactive proportion in specimens collected during 2020, of 1.2% compared to 0.7% in 2019 when SARS-CoV-2 was likely not circulating in the population. This suggests that a small number of the initially reactive samples collected in 2020 that failed to confirm on the alternate NAT assay may have been true positives, including the 2019 specimen that did not confirm positive but had positive antibody results.

The study data suggest an overall rate of RNAemia of about 1 per 1000 in the US deceased donor population during January through June 2020, and possible risk of viremia in donors from which tissues are recovered. Additionally, among the 17 initial NAT positive samples that were tested by serology, two were positive for anti-SARS-CoV-2 antibodies [one from 2019 NAT(−)/Ab(+) and one from 2020 NAT(+)/Ab(+)], suggesting that the remaining two of the confirmed NAT(+) donors likely had infections early in course of disease, before an antibody response could develop.

In comparison with blood donors tested using residual donor plasma minipools (MPs), we found a higher rate of RNAemia in deceased tissue donors, by a log factor of about 2 (100,000 vs 1000) (Bakkour et al. 2021). Testing with MPs may have resulted in lower detection amongst blood donors, particularly if any of the samples tested had low RNA concentrations (below ~ 100–300 copies/mL) (Bakkour et al. 2021). Blood donor communicable disease screening risk assessments are performed via first-person donor screening interviews and are considered more likely accurate compared to deceased donor screening risk assessments performed by obtaining information from others (e.g., next-of-kin). Furthermore, deceased donors sometimes have a poorly characterized cause of death; autopsies are not required. Estimates of RNAemia determined by blood donors reporting post donation information (PDI) indicative of respiratory illness (subsequent to the donation at a time when RNAemia is more likely to represent true viremia with replicating virus) during the pandemic suggests a higher rate of COVID-19 RNA detection compared with either the blood donor population or deceased tissue donors^13^ (Cappy et al. 2020).

Since the presence of RNA (RNAemia) does not imply transmissible live virus, the rate of true viremia among tissue donors is unknown but may be less than 1 in 1000. This is based on lack of infectious virus in COVID-19 patients with detected RNA in blood samples (Andersson et al. 2020). There were no samples available in our study for infectivity testing from the 3 confirmed samples. For two samples, quantity was not sufficient, and for the third, the sample was lost. The infectivity of blood and tissues where SARS CoV-2 RNA is detected is an area of active research and would be very helpful in considering tissue and organ transplantation risks.

Despite the potential risk that would be associated with viremia (if present), no transmission has been detected through blood or tissue products (Bakkour et al. 2021; Gaussen et al. 2021). Based on the current data, it is unlikely that SARS-CoV-2 is readily transmitted through tissue products, especially those that undergo processing. Studies have been performed for other respiratory viruses in blood donors, including influenza, and have not found evidence of transfusion transmission despite RNAemia (Likos et al. 2007; Dos Santos et al. 2020). Further studies examining the presence of RNA in unprocessed tissue recovered from SARS-CoV-2 nasal swab-positive donors are underway to provide tissue tropism information.

For solid organs, only lung transplantation has been proven to transmit SARS-CoV-2, with a possible, but unlikely, transmission via liver transplantation (Gaussen et al. 2021; Kaul et al. 2021; Heinz et al. 2020). Because donors in this study were anonymized, it is unknown how many of the study donors were also organ donors. Given the partial overlap between organ and tissue donors, the presence of RNAemia in organ donors should be considered possible. Upper respiratory samples were tested on all organ donors beginning around April 2020, and at the time no organ donors were accepted that had positive results for SARS CoV-2. While the significance of potential RNAemia is unknown, given there were 12,588 deceased organ donors in 2020 (Organ Procurement and Transplantation Network 2022) with such few transmissions, the data are reassuring. Now that some non-lung organ transplantation is being performed on SARS-CoV-2 positive organ donors, as part of the outcomes data collection there may be an opportunity to further investigate whether RNAemia or viremia is present at the time of transplantation (OPTN 2022).

There are important limitations to this study. The study necessarily used investigational assays under Emergency Use Authorization (EUA), and while the performance characteristics were not known, a recent peer-reviewed study has been published^11^. Performance characteristics of the assays on our study sample matrix, which were all collected after cessation of the heartbeat (from deceased donors, e.g.., “cadaveric” or “post-mortem” specimens), are still unknown. Furthermore, there were 134 samples that did not yield valid NAT screening results. The low rate (0.7%) of initial positive, unconfirmed results in 2019, when no virus was known to be circulating in the US, is reassuring concerning the specificity of the screening assay. Deceased donor specimen quality tends to be poor compared to those obtained from living or heart-beating individuals, and such factors as hemolysis, inhibitors, proteolysis, and hemodilution can affect assay results (Greenwald et al. 2018). The results may not be representative of the tissue donor population in the U.S., as results were obtained from a convenience sample of tissue banks who volunteered to participate. These results only apply to deceased tissue donors, given that samples tested were from deceased donor specimens—organ-only donors and living donors were not evaluated in this study. Furthermore, only donors that were *not* suspected of having COVID-19 were eligible for study participation, and rejected donors may have had a higher rate of RNAemia.

This study was made possible because of the willingness of tissue banks to participate, and due to the availability of study material because of voluntary policies regarding donor specimen storage. Funding was provided by the American Association of Tissue Banks (AATB), an industry trade organization, and in-kind contributions. There are no requirements by either AATB or the Food and Drug Administration (FDA) for storage of donor specimens. Furthermore, there are also no repositories of specimens from either tissue donors or recipients that are supported for research, unlike that which exists for blood donors, including National Heart, Lung, and Blood Institute’s Recipient Epidemiology and Donor Evaluation Study Program (REDS) (Kleinman et al. 2014), Centers for Disease Control and Prevention’s National Blood Donor Seroprevalence Study (NBDS) (Jones et al. 2021), and FDA’s Transfusion-Transmissible Infections Monitoring System (TTIMS) (Custer et al. 2016). We strongly encourage further research to include systematically evaluating the presence of SARS CoV-2 (and future emerging infectious diseases) in various tissues and organs, transmissibility studies of blood with SARS CoV-2 RNAemia, and provision of National Institutes of Health (NIH)-sponsored prospective specimen collection and storage from organ and tissue donors for use in research to inform donor evaluation in the face of future emerging infectious diseases. Such studies should be routinely considered early in the evaluation of emerging infectious diseases, as is currently done with blood donation.

In conclusion, SARS-CoV-2 RNA can be present in plasma and serum samples from deceased tissue donors, although incidence is low. The potential for transmission will be further elucidated with focused study on persistence of the virus in stored human tissue.

## Abbreviations

AATB: American Association of Tissue Banks
CI: Confidence interval
EUA: Emergency use authorization
MP: Minipools
NAT: Nucleic acid amplification test
NBDS: National Blood Donor Seroprevalence Study
NIH: National Institutes of Health
REDS: Recipient Epidemiology and Donor Evaluation Study Program
RUO: Research use only
TMA: Transcription-mediated nucleic acid amplification
TTIMS: Transfusion-Transmissible Infections Monitoring System
VRI: Vitalant Research Institute
